# The Role of Vitamin D in Postoperative Tendon Healing: A Scoping Review

**DOI:** 10.1177/23259671251371300

**Published:** 2025-10-14

**Authors:** Marc Daniel Bouchard, Danielle Dagher, Carl Keogh, Muath Alqahtani, Moin Khan

**Affiliations:** * McMaster University, Division of Orthopaedic Surgery, Hamilton, Canada; † McMaster University, Department of Health Research Methodology, Hamilton, Canada; ‡ Beaumont Hospital, Dublin, Ireland; § King Abdulaziz Medical City–National Guard Health Affairs, Jeddah, Saudi Arabia; ‖ Division of Orthopaedic Surgery, St. Joseph’s Hospital, Hamilton, Canada; Investigation performed at McMaster University, Hamilton, Canada

**Keywords:** tendon healing, vitamin D, rotator cuff, tendon repair

## Abstract

**Background:**

Tendon injuries pose significant clinical challenges because of poor vascularity and complex biomechanics, often leading to suboptimal healing. While surgical advancements and rehabilitation protocols have improved outcomes, delayed healing and high retear rates remain prevalent. Vitamin D, a key regulator of musculoskeletal health, may influence tendon healing through extracellular matrix remodeling, inflammation modulation, and cell proliferation. However, its effect on tendon repair remains unclear, with most studies focusing on rotator cuff tendons. This scoping review synthesizes current evidence on vitamin D’s role in tendon healing and identifies gaps in the literature.

**Purpose/Hypothesis:**

The purpose of this scoping review is to evaluate the available literature on vitamin D’s role in tendon healing and its influence on postoperative recovery. It was hypothesized that vitamin D deficiency is associated with poorer healing outcomes and increased failure rates.

**Study Design:**

Scoping review; Level of evidence, 4.

**Methods:**

A systematic search of Embase, OVID Medline, and Emcare databases was conducted through November 2024. Eligible studies included observational studies and randomized controlled trials assessing vitamin D status in adults undergoing tendon repair. Two independent reviewers screened and extracted data, resolving discrepancies with a third reviewer. Study quality was evaluated using the methodological index for non-randomized studies score. Given the heterogeneity of studies, results were synthesized narratively, focusing on evidence gaps across different tendons.

**Results:**

A total of 10 studies met inclusion criteria, with the majority focusing on rotator cuff repairs (n = 8). Vitamin D deficiency was consistently associated with poorer postoperative outcomes, including delayed healing, higher retear rates, and decreased functional recovery. Mechanistic studies suggested that vitamin D enhances healing through extracellular matrix remodeling and inflammation modulation. However, the overall quality of evidence was low, with most studies being retrospective. Notably, half of the studies relied on the same large administrative database, leading to potential patient overlap and limiting independent conclusions. Research on vitamin D’s role in healing other tendons, such as the Achilles, patellar, and flexor tendons, was lacking.

**Conclusion:**

Although evidence suggests vitamin D may support tendon healing, particularly in rotator cuff repair, studies remain low quality and limited to a single tendon group. Future prospective cohort studies and randomized controlled trials are needed to establish causality, assess its effects across various tendons, and determine optimal supplementation strategies.

Tendon injuries are a significant clinical challenge, affecting patients across a wide spectrum of activities and often requiring surgical repair to restore function. Despite advances in surgical techniques, the healing process for tendons remains suboptimal because of the inherently poor vascularity and complex biomechanical environment of tendon tissues.^18^ Recent studies suggest that vitamin D, a fat-soluble vitamin critical for calcium homeostasis and musculoskeletal health, may play a pivotal role in enhancing tendon repair and recovery.^20^

Vitamin D has long been recognized for its role in bone metabolism; however, emerging evidence points to its broader effect on connective tissue biology. Tendons express the vitamin D receptor, suggesting a direct biological role in modulating tendon physiology.^20^ Mechanistically, vitamin D influences processes essential for tendon healing, including cell proliferation, inflammation modulation, and extracellular matrix remodeling.^27^ Deficiencies in vitamin D are widespread, with over 1 billion people globally estimated to be affected.^2^ Such deficiencies have been associated with impaired musculoskeletal function and delayed healing after injuries.^10^ Numerous studies have reported low vitamin D levels in orthopaedic patients.^6,19^ This introduces a significant consideration, as research consistently demonstrates that individuals with higher vitamin D levels tend to exhibit improved muscle strength, power, and recovery capabilities.^3,4^ Furthermore, evidence suggests that vitamin D supplementation can enhance lower body strength, particularly in athletes, highlighting its potential role in optimizing physical performance and rehabilitation.^29^

The relationship between vitamin D and tendon healing is particularly relevant in rotator cuff injuries, which are among the most common tendon pathologies requiring surgical intervention. Rotator cuff repair strategies are adapted to the extent of tendon damage, ranging from minimally invasive arthroscopic procedures to more complex open surgical techniques. These approaches aim to restore function by securely reattaching the tendon to the humeral head, promoting optimal healing and biomechanical integrity.^9^ While surgical techniques for rotator cuff repair have been extensively studied, evidence specifically examining the role of vitamin D in tendon healing remains limited. The available literature is largely retrospective, often relying on overlapping patient populations and small sample sizes. Moreover, research on vitamin D’s role in healing for other tendons, such as the Achilles, patellar, and flexor tendons, is virtually nonexistent, creating a significant gap in knowledge.^25^ This scoping review aimed to evaluate the current evidence on the role of vitamin D in tendon healing. Additionally, it identified existing gaps in the literature across various tendon injuries and provides insights to guide future investigations and inform clinical strategies for optimizing tendon healing. We hypothesized that vitamin D deficiency would be associated with poorer tendon healing outcomes, including delayed recovery, higher retear rates, and decreased functional performance.

## Methods

### Search Strategy and Criteria

A scoping review was conducted in accordance with the PRISMA-ScR (Preferred Reporting Items for Systematic Reviews and Meta-Analyses extension for Scoping Reviews)^26^ to examine the role of vitamin D in tendon healing. A comprehensive search strategy was developed for the OVID Medline, Embase, and Emcare databases. Additional gray literature sources were searched to ensure a thorough and inclusive review of the evidence.^17^ Databases were searched from inception until November 21, 2024. Manual searches of reference lists of pertinent articles were also performed to ensure the inclusion of all relevant literature. For vitamin D, Medical Subject Headings (MeSH) terms such as *Vitamin D/* and *Vitamin D Deficiency/* were combined with free-text terms including "calciferol*" and "ergocalciferol*." Tendon-related concepts were captured using MeSH terms such as *Tendon Injury/* and *Tendon/* alongside terms such as "tendon rupture*," "tendon repair*," and "tendon heal*." Postoperative care and rehabilitation were identified using MeSH terms such as *Postoperative Care/*, *Postoperative Complications/*, *Rehabilitation/*, and *Treatment Outcome/*. Additional terms targeted functional recovery, including *Range of Motion/* and *Patient-Reported Outcome/*. The final search combined these sets using Boolean operators, narrowing results to studies addressing the intersection of vitamin D, tendon healing, and postoperative outcomes. The full search strategy is available in Appendix Table A1.

All search results were imported into the reference management software Covidence (Veritas Health Innovation Ltd) for deduplication and subsequent screening. For final article inclusion, 2 investigators (M.D.B. and C.K.) independently screened the titles, abstracts, and full-text articles resulting from the searches. Agreement between investigators was assessed with a kappa coefficient. A kappa statistic is a measure of interobserver variation. A grading system was used for the kappa coefficient whereby a kappa of 0.00 to 0.20 is considered slight agreement; 0.21 to 0.40 is considered fair agreement; 0.41 to 0.60 is considered moderate agreement; 0.61 to 0.80 is considered substantial agreement; and 0.81 to 1.00 is considered almost perfect agreement.^15^ Any disagreements were resolved through discussion with a third investigator and senior author (M.K.).

### Eligibility Criteria

Observational studies (cross-sectional, cohort, or case-control), randomized controlled trials (RCTs), and case series were eligible for inclusion. To be included, studies had to report vitamin D status of patients. Studies were considered to report appropriate outcomes if they assessed tendon healing, postoperative recovery, tendon pathology, or tendon-related functional outcomes in relation to vitamin D status. Studies were excluded for wrong outcomes if they focused solely on bone healing, general musculoskeletal health, or laboratory serum levels without clinical correlation. Appropriate study designs included observational studies (cohort, case-control, or cross-sectional) and RCTs involving adult human participants. Reviews, conference abstracts, animal studies, and studies without full-text availability were excluded as inappropriate designs. There were no restrictions on publication year; however, only studies published in English were considered.

### Methodological Assessment of Study Quality

The methodological index for non-randomized studies (MINORS) score was used to assess the quality of studies included in the review with a global ideal score of 16 for noncomparative studies and 24 for comparative studies.^24^ For this review, a score of ≤8 was considered to be of poor quality, 9 to 14 moderate quality, and 15 to 16 good quality for noncomparative studies (Appendix Table A2). These scores were ≤14, 15 to 22, and 23 to 24 for comparative studies, respectively. All included studies were assessed by 2 reviewers (M.D.B. and C.K.) who independently completed the risk of bias assessment; a third reviewer (M.K.) helped resolve any disagreement. There were no randomized studies identified that met the inclusion criteria.

### Data Collection and Abstraction

Data from eligible studies were extracted by the 2 independent reviewers using a standardized data collection form on Microsoft Excel (Version 16.90). The extracted data encompassed study characteristics (author, year of publication, level of evidence, study design, sample size, and database source), patient demographics (age and sex), and clinical variables (vitamin D status, affected tendon, and surgical details).

### Data Synthesis

Due to the heterogeneity of the included studies and overlap in the study populations, results were reported narratively, emphasizing key findings, emerging patterns, and gaps in the literature. Studies were categorized based on tendon type. Quantitative data, when available, were summarized descriptively. Descriptive statistics are reported as absolute frequencies with percentages or as means with corresponding measures of variance (standard deviation or range), where applicable.

## Results

### Literature Search

**Figure 1.**
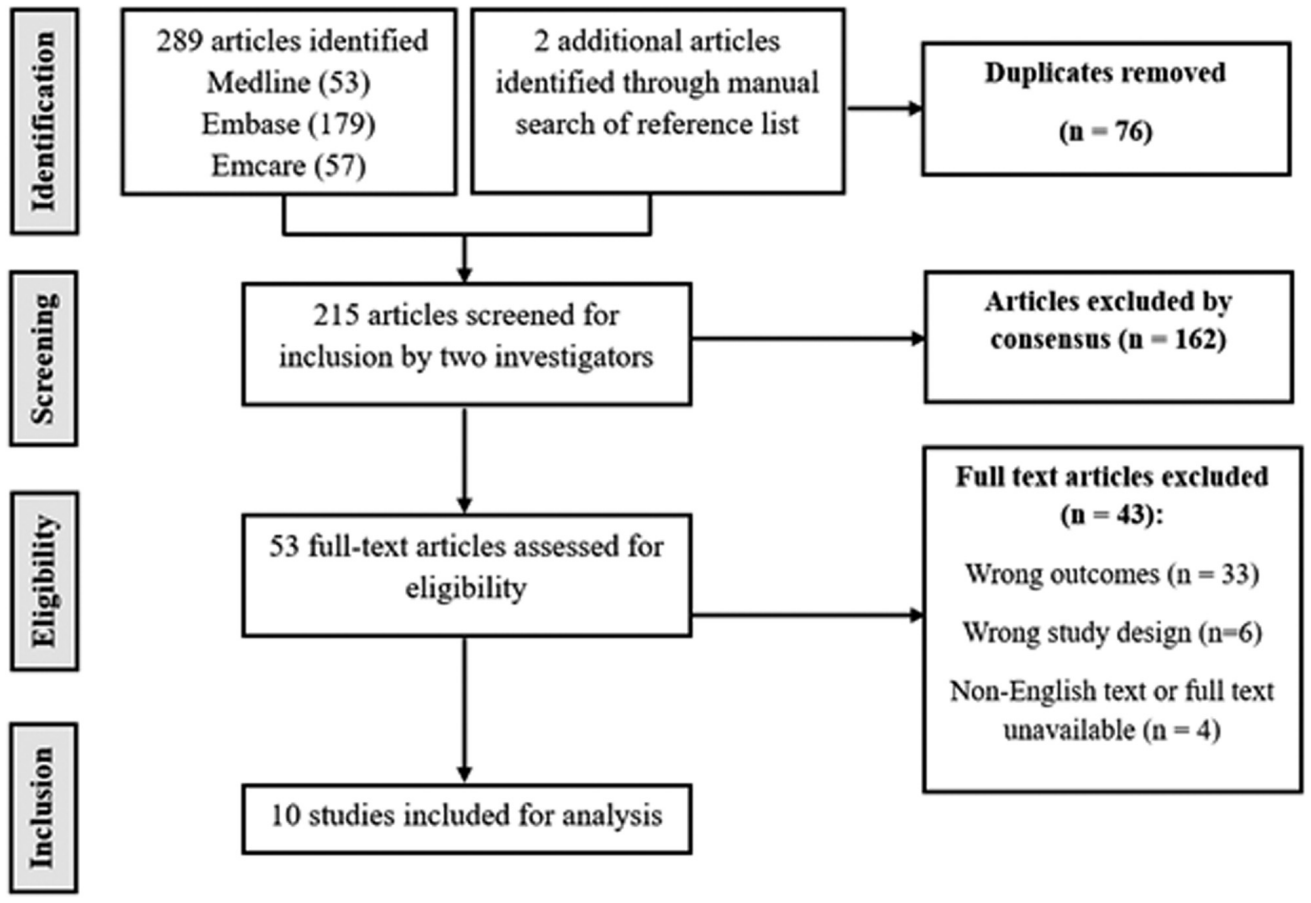
PRISMA (Preferred Reporting Items for Systematic Reviews and Meta-Analyses) flow diagram of study selection.

The database search identified 289 articles (see Appendix Table A1 for search strategies), with an additional 2 articles obtained through manual reference list searches. After removal of 76 duplicates, 215 articles remained for screening. Following title and abstract screening, 162 studies were excluded based on consensus between the 2 investigators. A total of 53 full-text articles were assessed for eligibility, with 43 studies excluded for the following reasons: wrong outcomes (n = 33), inappropriate study design (n = 6), and non-English or unavailable full text (n = 4). Ultimately, 10 studies met the inclusion criteria and were analyzed (Figure 1).

### Study Characteristics

Data on the characteristics of included studies are detailed in Table 1. Of the 10 studies included in this review, 7 comprised retrospective cohort studies,^1,7,8,13,16,23,28^ 1 prospective cohort study,^22^ 1 matched case-control study,^14^ and 1 case series.^21^ The majority of studies (8/10) were classified as level 3 evidence, while 1 study^21^ was level 4, and another^22^ was level 2.

**Table 1:** Study Characteristics and Demographics^*a*^

Author (year)	Study Design	Level of Evidence	Database Used	Sample Size	MINORS Score	Tendon Involved	Age, y^*b*^	Male, n (%)
Albright (2024)^1^	RCS	3	M151Ortho and MUExtr data sets within PearlDiver	336,320	4	Rotator cuff	55.7 ± 13.2	118,385 (35.2)
Cancienne (2019)^8^	RCS	3	PearlDiver	982	7	Rotator cuff	NR	340 (34.6)
Chen (2022)^7^	RCS	3	NR	89	8	Rotator cuff	61.2 ± 7.43	36 (40.4)
Harada (2019)^13^	RCS	3	PearlDiver	1881	6	Rotator cuff	NR	674 (35.8)
Khatri (2025)^14^	Matched case-control	3	PearlDiver	336,320	4	Biceps	55.7 ± 13.2	118,385 (35.2)
Lee (2021)^16^	RCS	3	NR	176	4	Rotator cuff	61.9 ± 8.90	82 (46.6)
O’Donnell (2020)^21^	Case series	4	PearlDiver	41,467	5	Rotator cuff	NR	21,853 (52.7)
Rhee (2023)^22^	PCS	2	NR	36	13	Rotator cuff	60.9 ± 9.20	15 (41.7)
Ryu (2015)^23^	RCS	3	NR	91	10	Rotator cuff	57.5 ± 4.30	47 (51.6)
Yaka (2022)^28^	RCS	3	Necmettin Erbakan University Meram Medicine Faculty	106	5	Lateral epicondyle	35.5 (range, 18-59)	50 (47.2)

a MINORS, methodological index for non-randomized studies; NR, not reported; PCS, prospective cohort study; RCS, retrospective cohort study.

b Data are presented as mean ± SD or mean (range).

Sample sizes ranged from 36 to 336,320. Five studies utilized the PearlDiver database,^1,8,13,14,21^ while others obtained data from institutional sources^28^ or did not report their database.^7,16,22,23^ Notably, the studies by Albright et al^1^ and Khatri et al^14^ analyzed the same patient cohort within the PearlDiver database, with the former investigating rotator cuff injuries and the latter focusing on biceps tendon injuries. The mean patient age, when reported, varied from 35.5 years to 61.9 years.

Most studies evaluated the rotator cuff tendons (n = 8; 80%). One study focused on the biceps tendon^14^ and another on the lateral epicondyle.^28^ The proportion of male patients ranged from 34.6% to 52.7%.

### Study Quality

All 10 studies included in this review were noncomparative. The total MINORS scores ranged from 4 to 13 out of a possible 16, with the majority being graded as poor quality.^1,7,8,13,14,16,21,28^ This was mostly owing to the absence of prospective data collection, loss to follow-up reporting, and unbiased endpoint assessment. Only 2 studies met the criteria for moderate quality,^22,24^ by these same standards (Appendix Table A2).

### Association Between Vitamin D Status and Tendon Pathology

All included studies assessed the relationship between vitamin D status and various tendon pathologies, with slight differences in classification criteria and outcomes evaluated (Table 2).

**Table 2:** Association Between Vitamin D Status and Tendon Pathology^*a*^

Author (year)	Database Used	Potential for Patient Sample Overlap	Vitamin D Status Classification	Key Findings
Rotator cuff				
Albright (2024)^1^	M151Ortho and MUExtr data sets within PearlDiver	Yes	Identified based on ICD-9-2689 and ICD-10-E559 codes	Vitamin D deficiency was associated with a greater than twofold risk of rotator cuff tears in the vitamin D cohort when compared with matched controls
Cancienne (2019)^8^	PearlDiver	Yes	Deficient: <20 ng/dl Insufficient: 20-30 ng/dl Sufficient: >30 ng/dl to <150 ng/dl	Vitamin D deficiency was associated with a significantly increased risk of revision rotator cuff surgery when compared with patients with normal vitamin D levels
Chen (2022)^7^	NR	NA	Deficient: <20 ng/dl Sufficient: >20 ng/dl	Preoperative vitamin D deficiency was associated with a higher retear rate and early pain after arthroscopic RCR
Harada (2019)^13^	PearlDiver	Yes	Deficient: <20 ng/dl Sufficient: >20 ng/dl	Vitamin D deficiency is associated with a greater risk of postoperative surgical complications and revision RCR after arthroscopic RCR
Lee (2021)^16^	NR	NA	Deficient: <20 ng/dl Insufficient: 20-30 ng/dl Sufficient: >30 ng/dl	The prevalence of vitamin D deficiency in patients with rotator cuff tears was 44.3%; age had a significant positive correlation with the serum concentration of vitamin D
O’Donnell (2020)^21^	PearlDiver	Yes	Identified based on ICD-9 codes	Vitamin D deficiency is an independent risk factor for failure of primary RCR requiring revision RCR
Rhee (2023)^22^	NA	NA	Deficient: <10 ng/dl Insufficient: 10-20 ng/dl Sufficient: >20 ng/dl	Lower serum vitamin D levels preoperatively and at 1 year after surgery were correlated with lower postoperative muscle power in abduction
Ryu (2015)^23^	NR	NA	Deficient: <20 ng/dl Insufficient: 20-30 ng/dl Sufficient: >30 ng/dl to <150 ng/dl Intoxicated: >150 ng/dl	Serum vitamin D level had no significant relationships with postoperative structural integrity and functional outcomes after arthroscopic RCR
Biceps				
Khatri (2025)^14^	PearlDiver	Yes	Identified based on ICD-9-2689 and ICD-10-E559 codes	Vitamin D deficiency increased the risk of distal biceps tendinopathy but did not increase the rate of surgical repair or revision
Lateral epicondyle				
Yaka (2022)^28^	Necmettin Erbakan University Meram Medicine Faculty	No	Deficient: <20 ng/dl Insufficient: 20-30 ng/dl Sufficient: >30 ng/dl	Low vitamin D may be one of the factors in the etiology of lateral epicondylitis

a ICD-9/ICD-10, International Classification of Diseases, Ninth Revision/Tenth Revision; NA, not applicable; NR, not reported; PCS, prospective cohort study; RCR, rotator cuff repair; RCS, retrospective cohort study.

Among the rotator cuff studies, 2 large database studies^1,21^ identified vitamin D deficiency using International Classification of Diseases (ICD), Ninth Revision (2689), and ICD, Tenth Revision (E559), codes, as specific serum concentrations were not available. Half of these studies^1,8,13,21^ used the PearlDiver database for patient identification, introducing the possibility for patient overlap. Five of these studies found an association between vitamin D deficiency and negative postoperative outcomes, including increased risk of rotator cuff tears, higher revision surgery rates, greater retear rates, and poorer functional recovery.^1,7,8,13,21^ Only 1 study found no significant relationship between vitamin D levels and rotator cuff repair outcomes.^23^

The study by Lee et al^16^ differed from the other rotator cuff studies, as it did not examine clinical outcomes but rather reported a 44.3% prevalence of vitamin D deficiency among patients with rotator cuff tears. Additionally, Rhee et al^22^ used a different vitamin D classification system than the other studies, defining deficiency as <10 ng/dL, rather than the more commonly used threshold of <20 ng/dL.

Beyond the rotator cuff, Khatri et al^14^ found an association between vitamin D deficiency and an increased risk of distal biceps tendinopathy, although it did not influence surgical outcomes. Yaka et al^28^ suggested a potential link between low vitamin D levels and lateral epicondylitis (Table 2).

## Discussion

This scoping review synthesizes the current evidence investigating the relationship between vitamin D status and tendon healing outcomes. Overall, the findings align with our original hypothesis, suggesting that vitamin D deficiency may contribute to poorer tendon healing and functional recovery. However, the strength of the evidence remains limited by methodological weaknesses across studies. Our review also identifies several important gaps in the literature, including an overwhelming focus on rotator cuff tendons, repeated reliance on overlapping patient databases, and a lack of high-quality, prospective research across diverse tendon types.

Several individual studies in this review suggested a potential benefit of vitamin D in postoperative tendon healing, with some reporting lower revision rates and favorable clinical outcomes in patients with sufficient vitamin D levels.^7,8,13,21,22^ Previous research has highlighted the role of vitamin D in musculoskeletal health, with studies demonstrating its involvement in collagen synthesis, extracellular matrix remodeling, and overall tendon homeostasis.^2,10,27^ Additionally, findings from related orthopaedic fields, such as fracture healing and muscle recovery, have further emphasized vitamin D’s importance in optimizing postinjury tissue repair.^5,12^ However, because of the significant limitations identified in this review, it remains challenging to draw definitive conclusions about the role of vitamin D in tendon healing after surgery.

One of the most striking findings is the overwhelming focus on rotator cuff tendons, with 8 out of 10 included studies exclusively examining rotator cuff repair outcomes.^1,7,8,13,16,21,22,23^ Only 2 studies investigated other tendons: one on lateral epicondylitis^28^ and another on biceps tendinopathy.^14^ There was no research exploring the role of vitamin D in the healing of other tendons such as flexor tendons, Achilles tendons, or patellar tendons. This lack of diversity in tendon types represents a major gap in the literature, as the pathophysiology and healing mechanisms of tendons vary significantly based on anatomic location and function. For instance, weightbearing tendons like the Achilles may have different healing responses compared with upper extremity tendons, given their biomechanical demands and vascularity.^11^ Previous studies have suggested that vitamin D plays a role in musculoskeletal health, particularly in the modulation of inflammation and extracellular matrix remodeling, which are essential processes in tendon healing.^2,10,27^ The predominance of rotator cuff studies may be attributed to the relatively high incidence of rotator cuff tears and the associated burden of poor postsurgical outcomes.^9,10,18^ However, the absence of data on other tendons limits the generalizability of findings and impedes the development of comprehensive guidelines for vitamin D supplementation in tendon healing across multiple anatomic sites.

Another significant methodological limitation identified in this review is the repeated use of the PearlDiver database across multiple studies, leading to potential patient cohort overlap. Five of the included studies utilized PearlDiver, raising concerns about redundancy in data analysis and the validity of cross-study comparisons. For example, both Albright et al^1^ and Khatri et al^14^ examined different tendon pathologies using the same PearlDiver cohort, which likely resulted in an overrepresentation of certain patient populations. This issue complicates statistical analyses and limits the ability to draw independent conclusions about vitamin D’s role in tendon healing. Additionally, the reliance on large administrative databases introduces inherent limitations related to retrospective study designs, lack of standardized vitamin D assessments, and incomplete clinical follow-up. Three out of the 10 studies in this review^1,14,21^ defined vitamin D deficiency based on ICD coding rather than serum concentration measurements, increasing the risk of misclassification bias.^20^ Furthermore, the absence of prospective data collection and standardized outcome measures weakens the reliability of reported associations between vitamin D levels and surgical success.

Another notable finding from this review is the association between vitamin D deficiency and biceps tendinopathy observed in the Khatri et al^14^ study. While this study did not increase the rate of surgical repair or revision, it suggests that vitamin D deficiency may contribute to the pathophysiology of tendinopathy. This aligns with previous research demonstrating the role of vitamin D in inflammation modulation and extracellular matrix remodeling, which are key factors in tendon degeneration.^25,27^ However, further studies are needed to determine whether vitamin D supplementation can mitigate tendinopathy progression or improve postsurgical healing outcomes in affected patients.

The overall quality of evidence in this field remains low, with most studies rated as poor based on the MINORS criteria and all of them being noncomparative. Of the 10 studies included in this review, only 1 (Rhee et al^22^) met the criteria for high quality owing to its prospective cohort design. The lack of RCTs represents a major gap in the literature, as RCTs provide the most robust evidence for establishing causation. Additionally, the sample sizes of many studies were relatively small, limiting the statistical power of their findings. Previous research has demonstrated that vitamin D supplementation can enhance muscle function and bone metabolism, but its direct effect on tendon healing remains unclear due to the methodological limitations of available studies.^3,4^

To address the current knowledge gaps, future studies should focus on expanding research beyond the rotator cuff to investigate vitamin D’s role in the healing of Achilles, flexor, patellar, and other tendons. Conducting prospective cohort studies and RCTs to establish causality between vitamin D levels and tendon healing outcomes will be essential. Standardized serum vitamin D assessments rather than ICD coding should be employed to ensure accurate deficiency classification. Moreover, reducing reliance on retrospective administrative databases and ensuring minimal patient overlap by diversifying data sources will improve study validity. Finally, investigating optimal vitamin D supplementation strategies, including dosage, timing, and duration, may provide clinical insights into enhancing postoperative tendon healing. It is important to recognize, however, that conducting large-scale prospective studies on vitamin D and tendon healing may be limited by funding constraints. Such studies would likely require unrestricted or philanthropic funding sources, given the challenges in securing traditional research grants for nutrient-focused musculoskeletal investigations.

## Conclusion

Consistent with our original hypothesis, the available evidence suggests an association between vitamin D deficiency and poorer postoperative tendon healing, although causality remains unproven due to the predominance of low-level evidence and a lack of high-level prospective research. Until more robust evidence is available, clinicians should consider individualized vitamin D assessments as part of perioperative care for tendon repair.

## Appendix

**Table A1:** Medical Subject Headings Terms and Boolean Operators Used to Develop Search Strategy From Embase, Emcare, and OVID Medline Databases

Database: Embase <1974 to 2024 November 20> Search Strategy:
1. exp Vitamin D/ or “vitamin D”.mp or exp Vitamin D Deficiency/ (214762)
2. calciferol*.mp or exp Ergocalciferols/ or ergocalciferol*.mp (10328)
3. 1 or 3 (214839)
4. exp Tendon Injury/ or “tendon injur*”.mp or “tendon rupture*”.mp (33499)
5. “tendon heal*”.mp or “tendon repair*”.mp or “tendon transplantation*”.mp or exp Tendon/ or tendon*.mp (128388)
6. 4 or 5 (134852)
7. exp Postoperative Care/ or exp Postoperative Complication/ or “postoperative recovery”.mp or “postoperative care”*.mp or postoperative.mp or “post-operative”.mp (1667201)
8. exp Rehabilitation/ or rehab*.mp (794603)
9. exp Treatment Outcome/ or outcome*.mp or “biomechanical strength”.mp or strength.mp (5751066)
10. “functional recovery”.mp or exp "Recovery of Function"/ or “range of motion”.mp or exp "Range of Motion"/ or “patient reported outcome measure”.mp or exp Patient-Reported Outcome/ (255704)
11. 7 or 8 or 9 or 10 (7296797)
12. 3 and 6 and 11 (179)
Database: Ovid Emcare <1995 to 2024 Week 47> Search Strategy:
1. exp Vitamin D/ or “vitamin D”.mp or exp Vitamin D Deficiency/ (49946)
2. calciferol*.mp or exp Ergocalciferols/ or ergocalciferol*.mp (1918)
3. 1 or 3 (49959)
4. exp Tendon Injury/ or “tendon injur*”.mp or “tendon rupture*”.mp (16201)
5. “tendon heal*”.mp or “tendon repair*”.mp or “tendon transplantation*”.mp or exp Tendon/ or tendon*.mp (47825)
6. 4 or 5 (51777)
7. exp Postoperative Care/ or exp Postoperative Complication/ or “postoperative recovery”.mp or “postoperative care”*.mp or postoperative.mp or “post-operative”.mp (400521)
8. exp Rehabilitation/ or rehab*.mp (349511)
9. exp Treatment Outcome/ or outcome*.mp or “biomechanical strength”.mp or strength.mp (1713531)
10. “functional recovery”.mp or exp "Recovery of Function"/ or “range of motion”.mp or exp "Range of Motion"/ or “patient reported outcome measure”.mp or exp Patient-Reported Outcome/ (106464)
11. 7 or 8 or 9 or 10 (2172658)
12. 3 and 6 and 11 (57)
Database: Ovid MEDLINE ALL <1946 to November 20, 2024> Search Strategy:
1. exp Vitamin D/ or “vitamin D”.mp or exp Vitamin D Deficiency/ (115309)
2. calciferol*.mp or exp Ergocalciferols/ or ergocalciferol*.mp (5166)
3. 1 or 2 (115390)
4. exp Tendon Injuries/ or “tendon injur*”.mp or “tendon rupture*”.mp (35347)
5. “tendon heal*”.mp or “tendon repair*”.mp or “tendon transplantation*”.mp or exp Tendons/ or tendon*.mp (102110)
6. 4 or 5 (107555)
7. exp Postoperative Care/ or exp Postoperative Complications/ or “postoperative recovery”.mp or “postoperative care”*.mp or postoperative.mp or “post-operative”.mp (1180440)
8. exp Rehabilitation/ or rehab*.mp (654192)
9. exp Treatment Outcome/ or outcome*.mp or “biomechanical strength”.mp or strength.mp (3970888)
10. “functional recovery”.mp or exp "Recovery of Function"/ or “range of motion”.mp or exp "Range of Motion, Articular"/ or “patient reported outcome*”.mp or exp Patient Reported Outcome Measures/ (213367)
11. 7 or 8 or 9 or 10 (5188127)
12. 3 and 6 and 11 (53)

**Table A2:** MINORS^*a*^ Score Calculations Quality Assessment of All Included Articles in Review

	Albright (2024)^17^	Cancienne (2019)^18^	Chen (2022)^19^	Harada (2019)^20^	Khatri (2025)^25^	Lee (2021)^21^	O’Donnell (2020)^26^	Rhee (2023)^24^	Ryu (2015)^22^	Yaka (2022)^23^
1. Clearly stated aim	2	2	2	2	2	2	2	2	2	2
2. Inclusion of consecutive patients	0	0	0	0	0	0	0	1	2	1
3. Prospective data collection	0	0	0	0	0	0	0	2	0	0
4. Endpoints appropriate to the aim of the study	2	2	2	2	2	2	2	2	2	2
5. Unbiased assessment of the study endpoint	0	0	1	0	0	0	0	2	1	0
6. Follow-up period appropriate to the aim of the study	0	2	2	1	0	0	1	2	2	0
7. Loss to follow-up <5%	0	1	1	1	0	0	0	1	1	0
8. Prospective calculation of the study size	0	0	0	0	0	0	0	1	0	0
9. Adequate control group	–	–	–	–	–	–	–	–	–	–
10. Contemporary groups	–	–	–	–	–	–	–	–	–	–
11. Baseline equivalence of groups	–	–	–	–	–	–	–	–	–	–
12. Adequate statistical analysis	–	–	–	–	–	–	–	–	–	–
Total MINORS score	4^*b*^	7^*b*^	8^*b*^	6^*b*^	4^*b*^	4^*b*^	5^*b*^	13^*c*^	10^*c*^	5^*b*^
Maximum possible score	16	16	16	16	16	16	16	16	16	16

a MINORS, methodological index for non-randomized studies.

b MINORS score, poor quality.

c MINORS score, moderate quality.

d MINORS score, good quality.
